# Additional saturday occupational therapy for adults receiving inpatient physiotherapy rehabilitation: a prospective cohort study

**DOI:** 10.1186/s12913-022-07727-7

**Published:** 2022-05-09

**Authors:** Erin L. Caruana, David Rowell, Suzanne S. Kuys, Sandra G. Brauer

**Affiliations:** 1 School of Health and Rehabilitation Sciences, The University of Queensland, Brisbane, Australia; 2 St Andrew’s War Memorial Hospital, Brisbane, Australia; 3 Centre for the Business and Economics of Health, The University of Queensland, Brisbane, Australia; 4 School of Allied Health, Australian Catholic University, Brisbane, Australia

**Keywords:** Multidiscipline, Rehabilitation, Economic evaluation

## Abstract

**Background:**

The first aim of this study was to investigate the impact of providing an additional four hours of Saturday occupational therapy to patients receiving Saturday physiotherapy in an inpatient setting on length of stay, functional independence, gait and balance. The second aim was to conduct an economic evaluation to determine if the introduction of a Saturday occupational therapy service in addition to physiotherapy resulted in a net cost savings for the rehabilitation facility.

**Methods:**

A prospective cohort study with a historical control was conducted in an Australian private mixed rehabilitation unit from 2015–2017. Clinical outcomes included the Functional Independence Measure (Motor, Cognitive, Total), gait speed (10 Meter Walk test) and five balance measures (Timed Up and Go test, Step test, Functional Reach, Feet Together Eyes Closed and the Balance Outcome Measure of Elder Rehabilitation). Economic outcomes were rehabilitation unit length of stay and additional treatment costs.

**Results:**

A total of 366 patients were admitted to the rehabilitation unit over two 20-week periods. The prospective cohort (receiving Saturday occupational therapy and physiotherapy) had 192 participants and the historical control group (receiving Saturday physiotherapy only) had 174 participants. On admission, intervention group participants had higher cognitive (*p* < 0.01) and total (*p* < 0.01) Functional Independence Measure scores. Participation in weekend therapy by the intervention group was 11% higher, attending more sessions (*p* < 0.01) for a greater length of time (*p* < 0.01) compared to the historical control group.

After controlling for differences in admission Functional Independence Measure scores, rehabilitation length of stay was estimated to be reduced by 1.39 (*p* = 0.08) days**.** The economic evaluation identified potential cost savings of AUD1,536 per patient. The largest potential savings were attributed to neurological patients AUD4,854. Traumatic and elective orthopaedic patients realised potential patient related cost savings per admission of AUD2,668 and AUD2,180, respectively.

**Conclusions:**

Implementation of four hours of Saturday occupational therapy in addition to physiotherapy results in a more efficient service, enabling a greater amount of therapy to be provided on a Saturday over a shorter length of stay. Provision of multidisciplinary Saturday rehabilitation is potentially cost reducing for the treating hospital.

**Supplementary Information:**

The online version contains supplementary material available at 10.1186/s12913-022-07727-7.

## Background

Rehabilitation aims to improve the functional status of people with health conditions leading to impairments, activity limitations or participation restrictions [1]. Multidisciplinary rehabilitation (including physical therapy (PT) and occupational therapy (OT)) optimizes patient outcomes, and is beneficial for geriatric patients [2], people following hip fracture [3, 4] and those with Parkinson’s Disease [3] amongst other conditions. The Australasian Faculty of Rehabilitation Medicine [5] and the Consultative Committee on Private Rehabilitation [6] recommends that rehabilitation should extend to weekends.

Randomised controlled trials have shown improvement in functional independence and health related quality of life with Saturday PT only [7] and for multidisciplinary (OT and PT) rehabilitation [8] compared to Monday to Friday rehabilitation. Additionally, although reductions in hospital length of stay (LOS) of 3.2 days (95% CI -0.5 to 6.9) have been reported for the PT only intervention [7] and 2.0 days (95% CI 0–4; *p* = 0.1) for the multidisciplinary intervention [8]; these findings have not always been statistically significant. However, the external validity of randomised controlled trials that strictly control intervention delivery could be questioned [9]. More recently, a systematic review combining weekend rehabilitation delivered over six and seven days has highlighted reductions in LOS [10]. One reason for the inconsistent effect on reduction in LOS maybe in the referral of a heterogeneous mix of patients for rehabilitation. Pragmatic implementation of a Saturday OT and PT rehabilitation service in an inpatient clinical setting would likely prioritise patients likely to benefit from [11–13] and be motivated to engage in additional rehabilitation [14, 15].

Providing weekend rehabilitation will likely incur additional staffing, hospital overhead and ward costs for facilities. However, economic evaluations have suggested that multidisciplinary Saturday rehabilitation may reduce costs per quality-adjusted life year gained [16, 17] with potential reductions also found in incremental cost-effectiveness ratios at 30 days [16] and 12 months [17] post discharge. The aims of this study were two-fold. Firstly, this study aimed to evaluate the efficacy of a pragmatic implementation of four hours of Saturday OT in addition to PT in an inpatient setting on length of stay, functional independence, gait and balance. The second aim was to conduct an economic evaluation to determine if the introduction of a Saturday OT service in addition to PT resulted in a net cost savings for the rehabilitation facility.

## Methods

### Participants

A pragmatic prospective cohort study with historical control was performed on all patients admitted for rehabilitation at St Andrew’s War Memorial Hospital, a privately funded hospital located in Brisbane, Australia. Australia has a dual network of public and private hospitals. Public hospitals are managed and funded by government offering free services to eligible people. Private hospitals provide fee for service care including care provided by medical and supplementary ancillary services including OT and PT [18]. Private hospitals more frequently provide weekend rehabilitation services [14]. In total, 366 patients admitted to a 20-bed rehabilitation ward were included; 174 patients admitted from October 2015 to April 2016 were the control group and 192 patients admitted from October 2016 to April 2017 were the intervention group. Ethical approval was granted by UnitingCare Health Human Research Ethics Committee (HREC#2014000752; 2011.16.38) and conformed to the Helsinki Declaration. Individual patient consent to participate in the study was not required by the ethics committee as the service was deemed usual practice.

### Intervention

The rehabilitation unit serviced a mixed adult caseload. Participants in both groups received usual weekday (Monday to Friday) rehabilitation consisting of nursing, medical, and individualised OT and PT (one hour each, per weekday) care, with speech pathology and dietetic involvement as required. A Saturday PT rehabilitation service consisting of 3.5 h was available for the unit. The Saturday PT rehabilitation could be delivered as group or individual sessions in the therapy gym or ward and was staffed by a PT (3.5 h) and an Assistant-in-Nursing (3 h) who provided porterage and assisted the PT as required, as no additional allied health assistant coverage was available. Control group participants were deemed eligible by their treating physiotherapist if they were clinically assessed to be likely to deteriorate over the weekend without PT input, were making functional improvements and would benefit from weekend PT input, were admitted on a Thursday or Friday, or admitted for a stay of less than one week. Patients were excluded from Saturday therapy if they consistently refused usual weekday PT or did not meet the inclusion criteria. Total therapy time available in the control period was 6.5 h each Saturday (3.5 PT hours, 3 h assistant/porterage hours).

The intervention group were offered a Saturday OT service in addition to the PT service, consisting of four hours each of OT and PT, with an allied health assistant providing porterage, therapy assistance and cleaning associated with therapy spaces. The Saturday PT service was extended by half an hour in line with OT and allied health assistant service provision. No change was made to PT eligibility criteria. The intervention group were eligible to attend the Saturday OT service if they were admitted on a Friday, required an initial assessment (activities of daily living, cognitive or neurological assessment), required compression therapy, were neurological patients who would benefit from weekend OT, or required additional OT prior to discharge. A maximum of two activities of daily living assessments could be scheduled each Saturday. OT was provided in group or individual sessions, in the therapy gym or ward. Participants could receive both OT and PT Saturday services. Total therapy time available for the unit during the intervention period was 12 h each Saturday (4 PT hours, 4 OT hours, 4 allied health assistant hours).

### Data collection

Patient demographic data collected included age, sex, primary diagnosis, discharge destination, rehabilitation inpatient LOS and nine indicators of patient capability (clinical measures of functional independence, gait speed and balance), measured on admission and discharge to the rehabilitation unit. Functional independence was recorded using Functional Independence Measure (FIM) Motor (FIM_Motor_), Cognitive (FIM_Cognitive_) and Total (FIM_Total_) scores [19, 20]. Gait speed was measured using the 10 Meter Walk Test (10MWT) [21, 22] Five valid and reliable measures of balance with older populations were used: the Timed Up and Go (TUG) test, [23, 24] Step test [25, 26], Functional Reach, [27, 28] maximum Feet Together Eyes Closed (FTEC) test [29], and the Balance Outcome Measure of Elder Rehabilitation (BOOMER) [30, 31]. Distributions for the 9 dependent variables of interest are reported in Figure A.1 in the Additional file 1.

The economic evaluation was conducted with financial data obtained from St Andrew’s War Memorial Hospital’s human resources department to estimate the costs of providing 20 weeks of Saturday rehabilitation for both groups. Estimates of variable costs (e.g., wages) and fixed costs (e.g., hospital overheads & ward expenses) were included. Labour costs, which included allied health professionals who provided the rehabilitation treatments and nursing staffing who assisted with patient porterage and therapy in control period, were based on wage rates per hour (inclusive of weekend loading and on-costs). Estimates of average cost per bed-day published by the Hospital Pricing Authority [32] were used to monetise potential savings due to reduced LOS. All rehabilitation costs were collected in 2017 Australian dollars and adjusted to 2020 Australian dollars using the Australian consumer price index [33]. The cost analysis concluded with a Monte Carlo simulation with 1000 replications to test the robustness of the cost analysis.

### Statistical analysis

First, to explore the effect of a Saturday OT service in addition to a PT service on patient health at discharge, the following multivariate regression model was estimated:

1 $${Cap}_{DC}={\alpha }_{0}+{\alpha }_{1}{MSR+\alpha }_{3}fem+{\alpha }_{4}age+{\alpha }_{2}\mathbf{D}\mathbf{x}{+\varepsilon }_{i}$$

The dependent variable *Cap*_*DC*_ denotes one of nine indicators of patient capabilities measured at discharge, which were three measures of functional independence, gait speed and five indicators of balance. The explanatory variable of interest MSR (multidisciplinary Saturday rehabilitation) was a dummy variable that takes the value of one if the patient was enrolled in the intervention group and zero if enrolled in the control group. Controls for sex (= 1 if female) and age (years) were also included. The vector **D** consisted of a set of dummy variables that control for admitting diagnosis (neurology, amputation, musculoskeletal, orthopaedic-trauma, orthopaedic-elective, reconditioning) and ${\varepsilon }_{i}$ was a random error term. The null hypothesis: Saturday OT service in addition to a PT service has no effect on ${Cap}_{DC},{ (H}_{0}:{\alpha }_{1}=0$), was rejected if ${\alpha }_{1}$ had a *p*-value < 0.05. Specifications with continuous dependent variables were estimated using ordinary least squared and specifications with dependent variables that were count data were estimated using Poisson regression and the marginal effects (dy/dx) reported.

Second, to test the effect of receiving multidisciplinary Saturday OT service in addition to a PT service on rehabilitation LOS, the following multivariate regression was estimated, to isolate the impact of the intervention on LOS:

2 $$LOS={\alpha }_{0}+{\alpha }_{1}MSR+{\alpha }_{2}{FIM}_{Motor}A+{\alpha }_{3}{FIM}_{Cognitive}A+{\alpha }_{4}\mathbf{A}+{\alpha }_{5}{\mathbf{D}+\alpha }_{6}fem+{\alpha }_{7}age+{\varepsilon }_{i}$$

The dependent variable *LOS* was a count of the number of days the patient stayed in the rehabilitation unit, *MSR,* a binary variable that took the value of one if participants received multidisciplinary Saturday OT service in addition to a PT service. Variables, ${FIM}_{Motor}A$ *and* ${FIM}_{Cognitive}A$, were FIM sub-scales measured on admission to the rehabilitation unit. Two sets of binary variables for day of admission (A) and day of discharge (D) were included to control for the effect that day of admission may have on LOS. Sundays were the omitted reference category from the model. Controls for sex and age were included and ${\varepsilon }_{i}$ was a random error term. LOS were count data, and therefore Eq. 2 was estimated using Poisson regression and the marginal effects were reported.

Equation 3 specified a sub-analysis, which includes a set of dichotomous variables for medical diagnosis (neurology, musculoskeletal, orthopaedic-trauma, orthopaedic-elective, reconditioning) and their interactions with the binary treatment variable multidisciplinary Saturday rehabilitation (MSR). The interaction terms enabled the effect of the multidisciplinary Saturday OT and PT service to be differentiated by clinical diagnosis (*Dx*).

3 $$LOS={\beta }_{0}+{\beta }_{1}MSR+{\beta }_{2}{FIM}_{Cognitive}+{\beta }_{3}{FIM}_{Motor}+{\beta }_{4}{FIM}_{Total}{+ \beta }_{5}\mathbf{A}+{\beta }_{6}{\mathbf{D}+\beta }_{7}fem+{\beta }_{8}age+{\beta }_{9}Dx+{\beta }_{10}Dx*MSR+{\eta }_{i}$$

## Results

There was no statistically significant difference between the Saturday OT service in addition to PT (intervention) and Saturday PT service (control) groups in age, sex, medical diagnosis, acute inpatient care, or discharge destination (see Table 1). Table 1 presents the average number and duration of sessions undertaken by patients on a Saturday, as well as the average number of occasions of service completed by the Saturday service for both intervention and control groups. The percentage of patients attending Saturday rehabilitation was 11% (83% vs 72%, p < 0.01) greater for the intervention group compared to the control group. In the intervention period, six participants received only OT, 63 participants received only PT and 91 participants received both OT and PT on a Saturday during their rehabilitation stay. The intervention group attended 0.7 (p < 0.01) more Saturday sessions, received 72 (p < 0.01) more minutes of therapy and 7.1 (p < 0.01) more occasions of Saturday service during their rehabilitation stay, including 1.5 (*p* < 0.01) more occasions of PT service. While there was no statistically significant difference in LOS for the acute admission, the average LOS in the rehabilitation unit was 2.4 (*p* < 0.01) days less for the intervention group compared to the control group.

**Table 1:** Clinical and demographic data for intervention and control groups

Variable	Control *n* = 172	Intervention *n* = 192	*Tests of means*^*a*^*, proportions*^*b*^* & distributions*^*c*^
Age, mean (SD)	77.7 (12.9)	78.8 (10.6)	*-*1.2*; p* = 0.3^*a*^
Female, n	108	130	22; *p* = 0.3^b^
Diagnosis, n			*p* = 0.1^c^
Stroke	7	4	
Neurology	28	16	
Amputee	5	-	
Musculoskeletal	6	11	
Orthopaedic – Trauma	30	40	
Orthopaedic – Elective	35	47	
Reconditioning	63	74	
Discharge destination, n			*p* = 0.5^c^
Home	140	163	
Low Level Care	3	2	
High Level Care	16	14	
Transition care or another hospital ward	10	11	
Participants attending Saturday therapy, n [%]	126 [72.4%]	160 [83.3%]	[11%]; *p* < 0.01^b^
Saturday sessions attended, mean (SD)			
Total	1.6 (1.1)	2.3 (1.4)	0.7; *p* < 0.01^*a*^
Physiotherapy	1.6 (1.1)	1.6 (1.0)	0*; p* = 0.9^*a*^
Occupational therapy	n.a	0.7 (0.8)	n.a
Minutes in Saturday therapy, mean (SD)			
Total	85.1 (53.2)	119.6 (68.0)	-34.5; *p* = < 0.01^*a*^
Physiotherapy	85.1 (53.2)	82.2 (50.0	2.9; *p* = 0.6^*a*^
Occupational therapy	n.a	37.4 (39.3)	n.a
Occasions of Saturday service, mean (SD)			
Total	10.4 (1.2)	17.5 (3.2)	7.1; *p* < 0.01^*a*^
Physiotherapy	10.6 (1.1)	12.1 (1.9)	1.5; *p* < 0.01^*a*^
Occupational therapy	n.a	6.0 (1.8)	n.a
LOS in acute inpatient care, mean (SD)	11.9 (12.3)	11.1 (8.5)	*-0.8; p* = 0.5^*a*^
LOS in rehabilitation, mean (SD)	16.4 (11.2)	14.0 (7.0)	-2.4; *p* < 0.01^a^

Differences in means, proportions and distributions were determined using independent *t-tests*^*a*^, equality of proportions test^b^ and Mann–Whitney U-tests^c^, respectively

*Abbreviations*: *LOS* Length of stay, *n.a.* not applicable

Table 2 compares outcomes at admission and discharge for the control and intervention groups. On admission, the intervention group had higher motor (3.3, *p* = 0.06), cognitive (2.3, *p* < 0.01), and total (5.3, *p* = 0.01) FIM scores, as well TUG (6.7, *p* = 0.07), FR (5.6, *p* < 0.01), FTEC (2.8, *p* = 0.08) and BOOMER (2.64, *p* < 0.01) test scores compared to the control group. The last column (Table 2) reports the difference in the relative improvement for each measure of recovery. Participants in the intervention group had smaller increases in their motor (-3.0, *p* < 0.01), cognitive (-1.1, *p* < 0.01) and total (-3.9, *p* < 0.01) FIM scores when compared with the control group. The intervention group also had a larger decrease in their TUG test scores (7.9, *p* < 0.01) compared to the control group. There were no other statistically significant differences in the indicators of recovery between the control and intervention groups (see last column Table 2).

**Table 2:** Comparison of outcome measures for all participants

	Cont. Period		Interv. Period		Cont. Period	Interv. Period	Interv. Period – Cont. Period
**Variables**	ADM	DC	ADM	DC	DC—ADM	DC—ADM	
	Mean (SD)	Mean (SD)	Mean (SD)	Mean (SD)	Mean (SD)	Mean (SD)	t test Mean diff (*p* value)
***Independence***							
FIM_Motor_ [13-91]	53. 8 (17.5)	77.4 (13.3)	57.1 (14.7)	77.7 (13.3)	23.7 (11.1)	20.6 (10.0)	-3.0***
FIM_Cognitive_ [5-35]	28.9 (6.5)	31.0 (4.9)	31.2 (4.9)	32.2 (4.1)	2.1 (3.2)	1.0 (2.1)	-1.1***
FIM_Total_ [18–126]	82.7 (21.7)	108.5 (16.9)	88.0 (17.4)	109.9 (15.7)	25.8 (12.6)	21.9 (10.7)	-3.9***
***Gait***							
10MWT [m/s]	0.6 (0.3)	0.7 (0.3)	0.6 (0.3)	0.8 (0.3)	0.2 (0.2)	0.2 (0.2)	-0.004
***Balance***							
TUG test [s]	30.2 (27.7)	26.2 (29.0)	37.0 (28.3)	23. 9 (16.3)	-7.8 (13.3)	-15.8 (21.3)	7.9***
Step test [avg. n]	2.6 (4.2)	5.8 (5.7)	2.8 (4.2)	5.8 (4.9)	3.2 (3.9)	3.0 (3.6)	-0.2
FR test [cm]	6.2 (9.2)	13.7 (13.3)	11.8 (10.8)	18.1 (10.2)	6.7 (10.3)	5.3 (8.0)	-1.4
FTEC test [0–30]	11.2 (13.1)	19. 5 (13.6)	14.1 (14.1)	22.6 (11.8)	7.8 (12.6)	7.7 (12.2)	-0.1
BOOMER [0–16]	2.7 (4.4)	6.1 (5.6)	5.4 (4.3)	9.0 (4.2)	2.5 (3.8)	3.0 (2.7)	0. 5

*Abbreviations*: *FIM* Functional Independent Measure, *10MWT* 10 Meter Walk Test, *TUG* Timed Up and Go, *FR* Functional Reach, F*TEC* Feet Together Eyes Closed, *BOOMER* Balance Outcome Measure of Elder Rehabilitation. Levels of statistical significance are *** p < .01, ** p < .05, * p < *.1*

### Clinical efficacy

After controlling for differences in sex, age and medical diagnosis, some measures of patient function at discharge were marginally greater in the intervention group (see Table 3) compared to the control group. Intervention group participants scored one point better on the FIM_Cognitive_ (1.06, *p* = 0.02) but there was no difference in the FIM_Total_. The intervention group had a faster 10MWT (0.08, *p* = 0.04) and a slightly better BOOMER score (1.14, *p* = 0.09) compared to the control group.

**Table 3:** Regression results for indicators of patient capabilities at discharge

*Dependent variables on Discharge*	FIM_Motor_	FIM_Cognitive_	FIM_Total_	10MWT	TUG	Step Test	FR	FTEC	BOOMER
Estimation method	Poisson (dy/dx)	Poisson (dy/dx)	Poisson (dy/dx)	OLS	OLS	Poisson (dy/dx)	OLS	OLS	Poisson (dy/dx)
*Explanatory variables*									
Intervention (1/0)	-0.195	1.056**	0.871	.078**	-2.722	-0.427	1.927	1.3	1.142*
Female (1/0)	0.813	0.882*	1.769	-.196***	6.76**	-1.935***	-2.197	-1.443	-0.628
Age (years)	-0.159**	-0.070***	-0.226***	-.003*	.094	-0.047**	-.338***	-.103	-0.054**
Stroke (1/0)	-9.855	-4.624***	-14.522*	.488***	omitted	-0.686	14.118**	11.562**	-2.484
Neuro (1/0)	-5.262*	-1.603**	-6.902**	.258***	15.374**	-0.564	13.083***	17.593***	-1.322
Amputation (1/0)	-4.497	0.422	-4.097	omitted	omitted	-5.899***	omitted	omitted	-8.209***
Arthritis (1/0)	-6.483*	-2.205*	-8.778**	omitted	22.496**	-2.956***	11.411***	14.936***	-2.635**
Orthopaedic Traumatic (1/0)	-5.220***	-1.154*	-6.375***	.159**	12.928***	-1.066*	19.98***	23.591***	-0.677
Orthopaedic Elective (1/0)	2.658*	0.429	3.024*	.222***	7.1**	0.441	24.418***	26.112***	0.593
Reconstruction (1/0)	n.a	n.a	n.a	.248***	10.244**	n.a	21.251***	23.435***	omitted
Constant	n.a	n.a	n.a	.894***	3.767	n.a	24.121***	7.521	n.a
n	356	356	356	265	266	250	186	230	164
R^2^ or Pseudo R^2^^(a)^	0.026^(a)^	0.031^(a)^	0.031^(a)^	.164	0.058	0.092^(a)^	0.266	0.145	.013^(a)^

(1/0) denotes a binary variable (= 1 if true & = 0 if otherwise). All dependent variables measured on discharge from the rehabilitation ward

*Abbreviations*: *10MWT* 10 Meter Walk Test, *BOOMER* Balance Outcome Measure of Elder Rehabilitation, *dy/dx* Marginal effects, *FIM* Functional Independent Measure, *FR* Functional Reach, *FTEC* Feet Together Eyes Closed, *OLS* Ordinary least squares, *TUG* Timed Up and Go. Variables omitted because of collinearity. Levels of statistical significance are *** p < .01, ** p < .05, * p < *.1*

^a^denotes R^2^ or Pseudo R^2^

Table 4 reports the coefficients with robust standard errors and marginal effects obtained from Eq. 2 using Poisson regression. Conditional upon controls for FIM on admission, days of admission and discharge to the rehabilitation unit, age and sex, the intervention group was associated with statistically significant reduction in LOS compared to the control group. The marginal effect of the intervention on LOS was estimated to be a reduction of 1.39 days.

**Table 4:** Poisson regression, Coefficients and marginal effects LOS in Rehabilitation

	Model 2			Model 3		
	Coef	SE	dy/dx	Coef	SE	dy/dx
MSR (1/0)	-.096*	.052	-1.387	.044	.048	0.626
Female (1/0)	-.029	.059	-0.411	-.002	.031	-0.035
Age (years)	-.006**	.003	-0.093	-.006***	.001	-0.082
FIM_Cognitive_ on admission	-.002	.005	-0.031	.001	.003	0.009
FIM_Motor_ on admission	-.019***	.002	-0.267	-.018***	.001	-0.251
Discharged on Monday (1/0)	-.248*	.151	-3.291	-.206**	.092	-2.753
Discharged on Tuesday (1/0)	-.252*	.144	-3.378	-.217**	.09	-2.921
Discharged on Wednesday (1/0)	-.278*	.145	-3.689	-.205**	.091	-2.760
Discharged on Thursday (1/0)	-.38**	.148	-4.866	-.327***	.092	-4.231
Discharged on Friday (1/0)	-.479***	.167	-5.941	-.441***	.093	-5.500
Discharged on Saturday (1/0)	-.138	.233	-1.858	-.097	.121	-1.328
Admitted on Monday (1/0)	.198**	.085	3.012	.197***	.042	2.976
Admitted on Tuesday (1/0)	.094	.095	1.387	.063	.048	0.912
Admitted on Wednesday (1/0)	.067	.089	0.981	.06	.047	0.872
Admitted on Thursday (1/0)	.096	.14	1.449	.094	.105	1.409
Admitted on Friday (1/0)	-.006	.275	-0.086	.078	.157	1.159
Admitted on Saturday (1/0)	.029	.095	0.418	.044	.044	0.639
**Diagnosis**						
Neurology (1/0)	n.a	n.a	n.a	-.147**	.075	-1.989
Musculoskeletal (1/0)	n.a	n.a	n.a	-.444***	.128	-5.226
Orthopaedic-traumatic (1/0)	n.a	n.a	n.a	-.186**	.078	-2.505
Orthopaedic-elective (1/0)	n.a	n.a	n.a	-.395***	.08	-5.087
Reconditioning (1/0)	n.a	n.a	n.a	-.407***	.072	-5.550
**Interaction (Diagnosis x MSR)**						
Stroke * MSR (1/0)	n.a	n.a	n.a	-.148	.132	-1.965
Neurology * MSR (1/0)	n.a	n.a	n.a	-.366***	.096	-4.442
Musculoskeletal * MSR (1/0)	n.a	n.a	n.a	-.106	.147	-1.439
Orthopaedic-traumatic * MSR (1/0)	n.a	n.a	n.a	-.182**	.074	-2.418
Orthopaedic-elective * MSR (1/0)	n.a	n.a	n.a	-.146*	.082	-1.976
Reconditioning * MSR (1/0)	n.a	n.a	n.a	0		
Constant	4.58***	0.337	n.a	4.601***	.165	n.a
Pseudo R^2^	0.20			0.22		
Observations	365			365		

(1/0) denotes a binary variable (= 1 if true & = 0 if otherwise). The diagnosis “Stroke” omitted from Model 2 because of multi-collinearity

*Abbreviations Coef* Coefficient, *dy/dx* Marginal Effect, *FIM* Functional Independence Measure, *LOS* Length of Stay, *MSR* Multidisciplinary Saturday PT and OT Rehabilitation, *n.a*. Not Applicable, *SE* Standard error. Levels of statistical significance are *** p < .01, ** p < .05, * p < *.1*

### Economic evaluation

All relevant cost categories were captured. The principal cost category was wages; 85% and 90% of total costs for the control and intervention treatments, respectively (see Wage Costs Table 5). Our estimates did not include equipment depreciation and allocated floor space, though these are reported as minor in comparable economic analyses [16]. Approximately, 2.4% of the ward overheads were allocated to the interventions on the basis that the rehabilitation service was delivered in 4 of the 168 h that the ward was operational (see Ward Expenses Table 5).

**Table 5:** Costs for Saturday service for control and intervention groups

**Cost Categories**	Control Group (Nov 2015-March 2016) AUD2016	Intervention Group (Oct 2016-March 2017) AUD2016
**Wage Costs for Saturday Service**		
Physiotherapy (base rate)	$45	$46
Casual loading 25%	$11	$12
Hospital overheads 30%	$14	$14
Saturday loading 50%	$23	$23
Total per hour	$93	$95
Cost per week		
3.5 h/week in control group	$325	
4 h/week in intervention group		$381
Cost for 20 weeks	$6,503	$7,619
Registered Nurse for porterage (base rate)	$45	n.a
Casual loading 25%	$11	
Hospital overheads 30%	$14	
Saturday loading 50%	$23	
Cost per hour	$93	
Cost per week (2 h per week)	$185	
Cost for 20 weeks	$3,701	
Occupational therapist (base rate)	n.a	$47
Casual Loading 25%		$12
Hospital overheads 30%		$14
Saturday loading 50%		$23
Cost per hour		$95
Cost per week (4 h per week)		$381
Cost for 20 weeks		$7,619
Allied Health Assistant (base rate)	n.a	$28
Casual loading 25%		$7
Hospital overheads 30%		$8
Saturday loading 50%		$14
Cost per hour		$57
Cost per week (4 h per week)		$230
Cost for 20 weeks		$4,592
Wage operating costs allocated to intervention^a^	$1,932	$2,176
Total costs for Saturday service	**$12,137**	**$22,007**
**Ward operating costs**		
Housekeeping Supplies	$5,896	$5,475
Laundry Supplies	$37,160	$36,436
Printing & Stationery	$3,204	$5,677
Property Expenses	$9,940	$20,009
Marketing & Entertainment	$1,007	$0
Catering—Functions	$4,399	$2,633
Hospital Contractor Services^b^	$7,366	$7,145
Rates & Body Corporate^b^	$1,280	$1,313
Utilities^b^	$7,354	$6,755
Finance & Accounting^b^	$309	$344
Insurance^b^	$3,233	$3,436
Total ward overheads^b^	$81,147	$89,223

^a^2.4% of the total ward overheads were allocated to the intervention because the intervention consumed 4/168 h per week. ^b^Hospital overheads (0.82%) were allocated the operation of the rehabilitation ward. All costs reported as AUD2016 and rounded to the nearest dollar. Summary costs are adjusted to AUD2020 when transferred to Table 6

*Abbreviation n.a* not applicable

After adjustment to AUD2020, the costs of providing 20 weeks of rehabilitation to the control and intervention groups were estimated to be AUD12,784 and AUD23,180, respectively (Table 6). Controlling for confounding factors with the statistical models decreased the estimated reduction in LOS from 2.4 days (see Table 1) to 1.39 days (see Table 3). The latter estimate implies a total saving of 267 bed-days for the intervention group. Given a cost of AUD1,144 per rehabilitation bed-day [32, 33] the implied savings are AUD1,536 per patient (see Table 5 for details). A two-way sensitivity analysis of the parameters reduced LOS (1.39 ± 0.77) and cost per bed-day (AUD1,144 ± 305), using Monte Carlo simulation (*n* = 1,000) indicated that the monetised value of reduced LOS was greater than the costs of providing additional OT on a Saturday in approximately 95% of simulations.

**Table 6:** Cost Analysis; Multidisciplinary Saturday rehabilitation

Parameters	Values (AUD2020)
Patients in intervention group	192
Reduction in mean LOS, days (mean ± SD)	1.39 ± 0.77
Reduction in total LOS for Intervention group, (days)	266.9
Cost per rehabilitation bed-day, (AUD, mean ± SD)[32, 33]	$1,144 ± $305
Total savings (Cost per bed-day x Reduction in total LOS)	$305,328
Costs for Saturday Rehabilitation	
Intervention group	$23,180
Control group	$12,784
Net Cost (Intervention – Control)	$ 10,396
Net Savings (Total savings – Net cost)	294,932
Net Savings per patient	$1,536

Cost per rehabilitation bed-day was reported in AUD(201)4 [32] using DRG code Z60Z from “Cost weights for AR-DRG Version 7.0 Round 18 (2013–14) Public Sector Sample DRG” (mean cost / mean LOS) and adjusted to 2020 Australian dollars (AUD) using the Australian consumer price index [32, 33]. Treatment costs reported as AUD(2016) in Table 5 were adjusted to AUD(2020) in Table 6 using the Australian consumer price index [32] *Abbreviations*, *LOS* length of stay, *SD* standard deviation

Model 3, in Table 4, is a sensitivity analysis, which includes a set of binary variables that interacted medical diagnoses with Saturday OT and PT service, found that within the treatment group only neurological and orthopaedic patients had a statistically significant reduction in LOS. The marginal effect for neurological patients was a reduction of 4.4 days (Table 3). Hence the implied cost savings for patients with a neurological diagnosis is AUD4,854 per treated patient. Both traumatic and elective orthopaedic patients also benefited from Saturday OT and PT rehabilitation service with a reduced LOS resulting in implied cost savings of AUD2,668 and AUD2,180 per patient, respectively.

## Discussion

This paper used a pragmatic prospective cohort study design to analyse the effect of Saturday OT service in addition to a PT service on patient outcomes admitted to a 20-bed rehabilitation ward in a private hospital located in Brisbane, Australia. The aim was two-fold. The first to analyse the impact on LOS and functional status, and second to conduct an economic evaluation from the perspective of the healthcare provider. Outcome measures of functional status included functional independence, gait and balance. LOS and hospital cost data were obtained for the economic evaluation.

Controlling for age, sex and admitting diagnosis identified minor improvements in cognition and the composite balance measure (BOOMER) scores. After controlling for admission FIM, age, sex and days of admission and discharge, LOS for the intervention group was estimated to be 1.39 days less than the control group. This estimate corroborates results published by earlier research [7, 8]. Published costs per rehabilitation bed-day [32], and treatment cost estimates obtained from the hospital billing accounting department, identified potential cost savings of AUD1,536 per patient in the intervention group. This Saturday rehabilitation service consisting of OT and PT met the published Standards for the Provision of Inpatient Adult Rehabilitation Medicine Services in Public and Private Hospitals [5] providing rehabilitation a minimum of five days per week. However, the service did not meet the Guidelines for Recognition of Private Hospital Based Rehabilitation Services [6] which state that specialist rehabilitation services should be provided seven days per week [6]. While the benefits of additional rehabilitation services outside of usual business hours seems established, [34] Australian guidelines provide inconsistent advice for service providers. Providing rehabilitation therapy across six days (at least in stroke populations) appears to result in better patient outcomes compared to seven-day rehabilitation, [35] though few studies have specifically investigated these models. Additionally, providing rehabilitation across six days seems to be prevalent in Australian rehabilitation facilities [14, 36]. This current study adds to the evidence that rehabilitation six days a week is beneficial for patients and service providers alike.

Interestingly, greater reductions in LOS have previously been found with facilities providing PT [7, 37] compared to multidisciplinary weekend services [8, 13, 38, 39]. While this current study reports similar reductions in LOS as another Australian study [8] also providing Saturday OT and PT rehabilitation, the Saturday OT and PT rehabilitation service provision model warrants further pragmatic investigation to determine if these results are reproducible in different service models and settings. This reduction in LOS may have implications, not just for patient outcomes and health service costs, but also in terms of improved patient flow through both rehabilitation units and hospitals. Certainly, allied health managers perceive improved patient flow and quality of care are benefits associated with weekend services, at least in acute care [40]. An associated increase in throughput occurred in this rehabilitation unit with approximately 10% more patients admitted during the intervention period compared to the control period. This may have led to an improved flow of patients through the hospital and possibly reduced rehabilitation waiting lists.

Participants in the intervention group in this current study had higher scores on some measures of functional independence, and some balance measures on admission. It is unclear why this was the case. The participating facility had no change in admission criteria or admission processes. Our regression models for LOS (Table 4) did control for FIM_Motor_ on admission but did not explicitly control for other measures of balance. It is therefore possible that our estimates slightly overestimated reduced LOS. At discharge, largely both groups had similar functional independence, balance and gait and both groups improved performance met minimum clinical important differences. It is reasonable to suggest that discharge is likely determined by patient readiness, functional performance, and preparedness of the home environment [40]. Previous studies have reported similar discharge function from inpatient rehabilitation [11, 41]. Interestingly differences in cognitive function were noted between the two groups at discharge. The intervention group had better cognitive function at discharge compared to the control group, though both groups’ scores suggest discharge home would be likely. Results obtained from observational data are always subject to the *ceteris paribus* caveat, and causal inferences should be drawn with caution. Although our statistical models have controlled for some important observed differences between the control and the intervention groups, it is always possible that unobserved differences could confound our results.

Potential cost savings identified in the economic evaluation corroborate an evolving literature that suggests the provision of weekend rehabilitation services may deliver an economic dividend [7, 8, 16, 17]. Previous randomised controlled trials have reported that weekend rehabilitation may reduce hospital LOS [7, 8]. A cost utility analysis has also reported probable cost effectiveness at 30 days [16] and 12 months [17] post-discharge. We found that rehabilitation LOS reduced on average by 1.39 days, with long-stay inpatients appearing to benefit most from the intervention. It is worth noting that patients in the intervention group experienced a shorter length of stay and demonstrated smaller improvements in most functional measures compared to those in the control group. In the context of the economic evaluation conducted in the current study, this suggests that the potential cost savings may be at the expense of functional improvement. However, it is likely that patients in both groups were discharged when a minimum threshold for safe discharge home, of approximately 110 on the FIM, was met. This reduced functional gain in the intervention group is likely due to higher admission FIM scores compared to the control group. Diagnosis also appeared to be important with sub-analyses confirming larger LOS reductions for neurological and orthopaedic patients. It is perhaps not surprising that those who stay longer and have complex conditions would show greater benefit from the additional therapy offered through a Saturday OT and PT rehabilitation service compared to a PT only service; perhaps further validating the need for this service.

### Limitations

This study was conducted in a private health service in Brisbane, Australia and as such findings may not be generalisable to other settings including publicly funded health services. Although private hospitals more commonly provide weekend rehabilitation services than publicly funded facilities [13] we believe this study provides evidence to support implementation in other contexts. Assessors were not blind to group allocation as the Saturday rehabilitation was considered usual care, however staff were not aware of the focus of the study at the time of data collection, thus minimising the potential for assessor bias.

While utilizing hospital administration data to obtain individual patient level cost data can provide accurate cost estimates, this approach is resource intensive and was not a feasible option for this study. Instead, reduced LOS was used as a measure of the cost of treatment savings. However, a potential limitation of using the average cost of a rehabilitation bed-day as a proxy for the marginal cost of a rehabilitation bed-day is that this can result in an overestimation of cost-savings when the cost of the final day of admission is substantially less than the cost of an average bed-day [42]. This can frequently occur with acute inpatient admissions. However, our cost modelling has assumed that the costs of a rehabilitation bed-day did not significantly decline over the duration of the admission to rehabilitation ward. Furthermore, the extent to which a reduction in LOS will generate actual “savings” to the hospital will be determined by the funding model. If patients were funded *per diem* the benefit to the hospital would manifest as an improved throughput for the same cost. Alternatively, if the hospital was funded via a prospective payment model based on the admitting diagnosis the hospital would reduce their operating costs per treated patient.

A final limitation was that this economic evaluation was restricted to the perspective of the healthcare provider and hence we have reported the results of a costs-analysis rather than a cost-effectiveness analysis. If our economic evaluation was conducted from a societal perspective, the inclusion of improvements in FIM and BOOMER scores would have captured benefits in patient health status not currently included in this analysis.

## Conclusion

The provision of a multidisciplinary Saturday rehabilitation service comprising OT and PT leads to a greater reduction in LOS compared a to a Saturday PT service, even when controlling for discrepancies in admission function. Providing a Saturday OT and PT rehabilitation service resulted in more patients receiving weekend therapy compared to Saturday physiotherapy only rehabilitation. The provision of a Saturday OT and PT rehabilitation service is potentially cost reducing for the treating hospital.

## Supplementary Information

**Additional file 1: Figure A1.**Distributions for nine measures of patient capability at discharge.

## Data Availability

The datasets used and/or analysed during the current study are available from the corresponding author on reasonable request.

## Abbreviations

10MWT: 10-Meter Walk Test
AUD: Australian dollar
BOOMER: Balance Outcome Measure for Elder Rehabilitation
FIM: Functional Independence Measure
FTEC: Feet together eyes closed
LOS: Length of stay
MSR: Multidisciplinary Saturday rehabilitation
OT: Occupational Therapy
PT: Physical Therapy
TUG: Timed Up and Go

## References

[1] Australian Institute of Health and Welfare (2019). AIoHa. Admitted patient care 2017–18: Australian hospital statistics.

[2] Bachmann S, Finger C, Huss A, Egger M, Stuck AE, Clough-Gorr KM (2010). Inpatient rehabilitation specifically designed for geriatric patients: systematic review and meta-analysis of randomised controlled trials. BMJ..

[3] Halbert J, Crotty M, Whitehead C, Cameron I, Kurrle S, Graham S (2007). Multi-disciplinary rehabilitation after hip fracture is associated with improved outcome: A systematic review. J Rehabil Med.

[4] Taraldsen K, Sletvold O, Thingstad P, Saltvedt I, Granat MH, Lydersen S (2014). Physical behavior and function early after hip fracture surgery in patients receiving comprehensive geriatric care or orthopedic care–a randomized controlled trial. J Gerontol A Biol Sci Med Sci.

[5] Australasian Faculty of Rehabilitation Medicine (2019). Standards for the provision of inpatient adult rehabilitation medicine services in public and private hospitals 2019.

[6] Consultative Committee on Private Rehabilitation (2016). Guidelines for recognition of private hospital-based rehabilitation services.

[7] Brusco NK, Shields N, Taylor NF, Paratz J (2007). A Saturday physiotherapy service may decrease length of stay in patients undergoing rehabilitation in hospital: a randomised controlled trial. Aust J Physiother.

[8] Peiris CL, Shields N, Brusco NK, Watts JJ, Taylor NF (2013). Additional Saturday rehabilitation improves functional independence and quality of life and reduces length of stay: a randomized controlled trial. BMC Med.

[9] Rothwell PM (2005). External validity of randomised controlled trials:“to whom do the results of this trial apply?”. Lancet.

[10] Sarkies MN, White J, Henderson K, Haas R, Bowles J, Evidence Translation in Allied Health G (2018). Additional weekend allied health services reduce length of stay in subacute rehabilitation wards but their effectiveness and cost-effectiveness are unclear in acute general medical and surgical hospital wards: a systematic review. J Physiother.

[11] Caruana EL, Kuys SS, Clarke J, Brauer SG (2018). The impact of staffing model in a 6-day rehabilitation physiotherapy service. Physiother Res Int.

[12] Caruana EL, Kuys SS, Clarke J, Brauer SG (2019). Implementing a 6-day physiotherapy service in rehabilitation: exploring staff perceptions. Aust Health Rev.

[13] Hakkennes S, Lindner C, Reid J (2015). Implementing an inpatient rehabilitation Saturday service is associated with improved patient outcomes and facilitates patient flow across the health care continuum. Disabil Rehabil.

[14] Caruana EL, Kuys SS, Brauer SG (2018). Allied health weekend service provision in Australian rehabilitation units. Australas J Ageing.

[15] Caruana EL, Kuys SS, Clarke J, Bauer SG (2017). A pragmatic implementation of a 6-day physiotherapy service in a mixed inpatient rehabilitation unit. Disabil Rehabil.

[16] Brusco NK, Watts JJ, Shields N, Taylor NF (2014). Are weekend inpatient rehabilitation services value for money? An economic evaluation alongside a randomized controlled trial with a 30 day follow up. BMC Med.

[17] Brusco NK, Watts JJ, Shields N, Taylor NF (2015). Is cost effectiveness sustained after weekend inpatient rehabilitation? 12 month follow up from a randomized controlled trial. BMC Health Serv Res.

[18] Australian Institute of Health and Welfare (2020). Australia’s hospitals at a glance 2018–19. Cat. no. HSE 247.

[19] Ottenbacher KJ, Hsu Y, Granger CV, Fiedler RC (1996). The reliability of the functional independence measure: a quantitative review. Arch Phys Med Rehabil.

[20] Passalent LA, Tyas JE, Jaglal SB, Cott CA (2011). The FIM™ as a measure of change in function after discharge from inpatient rehabilitation: a Canadian perspective. Disabil Rehabil.

[21] Wolf SL, Catlin PA, Gage K, Gurucharri K, Robertson R, Stephen K (1999). Establishing the reliability and validity of measurements of walking time using the Emory Functional Ambulation Profile. Phys Ther.

[22] Bohannon RW (1997). Comfortable and maximum walking speed of adults aged 20–79 years: reference values and determinants. Age Ageing.

[23] Podsiadlo D, Richardson S (1991). The timed “Up & Go”: a test of basic functional mobility for frail elderly persons. J Amer Geriatr Soc.

[24] Steffen TM, Hacker TA, Mollinger L (2002). Age-and gender-related test performance in community-dwelling elderly people: Six-Minute Walk Test, Berg Balance Scale, Timed Up & Go Test, and gait speeds. Phys Ther.

[25] Hill KD, Bernhardt J, McGann AM, Maltese D, Berkovits D (1996). A new test of dynamic standing balance for stroke patients: reliability, validity and comparison with healthy elderly. Physiother Can.

[26] Hong S-J, Goh EY, Chua SY, Ng SS (2012). Reliability and validity of step test scores in subjects with chronic stroke. Arch Phys Med Rehabil.

[27] Duncan PW, Weiner DK, Chandler J, Studenski S (1990). Functional reach: a new clinical measure of balance. J Gerontol.

[28] Isles RC, Choy NLL, Steer M, Nitz JC (2004). Normal values of balance tests in women aged 20–80. J Amer Geriat Soc.

[29] Cohen H, Blatchly CA, Gombash LL (1993). A study of the clinical test of sensory interaction and balance. Phys Ther.

[30] Haines T, Kuys SS, Morrison G, Clarke J, Bew P, McPhail S (2007). Development and validation of the balance outcome measure for elder rehabilitation. Arch Phys Med Rehabil.

[31] Kuys SS, Morrison G, Bew PG, Clarke J, Haines TP (2011). Further validation of the balance outcome measure for elder rehabilitation. Arch Phys Med Rehabil.

[32] Independent Hospital Pricing Authority (2015). Australian public hospitals cost report 2013–2014 round 18.

[33] Australian Bureau of Statistics (ABS). 6202.0 Labour Force, Australia Table 1. Labour force status by Sex, Australia - Trend, Seasonally adjusted and Original 2018 [Cited 20 March 2018]. Available from: http://www.abs.gov.au/AUSSTATS/abs@.nsf/DetailsPage/6202.0Feb%202018?OpenDocument.

[34] Scrivener K, Jones T, Schurr K, Graham PL, Dean CM (2015). After-hours or weekend rehabilitation improves outcomes and increases physical activity but does not affect length of stay: a systematic review. J Physiother.

[35] English C, Shields N, Brusco NK, Taylor NF, Watts JJ, Peiris C (2016). Additional weekend therapy may reduce length of rehabilitation stay after stroke: a meta-analysis of individual patient data. J Physiother.

[36] Shaw KD, Taylor NF, Brusco NK (2013). Physiotherapy services provided outside of business hours in Australian hospitals: a national survey. Physiother Res Internat.

[37] English C, Bernhardt J, Crotty M, Esterman A, Segal L, Hillier S (2015). Circuit class therapy or seven-day week therapy for increasing rehabilitation intensity of therapy after stroke (CIRCIT): a randomized controlled trial. Internat J Stroke.

[38] DiSotto-Monastero M, Chen X, Fisch S, Donaghy S, Gomez M (2012). Efficacy of 7 days per week inpatient admissions and rehabilitation therapy. Arch Phys Med Rehabil.

[39] Ruff RM, Yarnell S, Marinos JM (1999). Are stroke patients discharged sooner if in-patient rehabilitation services are provided seven v six days per week?. Amer J Phys Med Rehabil.

[40] Mitchell D, O’Brien L, Bardoel A, Haines T (2017). Challenges, uncertainties and perceived benefits of providing weekend allied health services—a managers’ perspective. BMC Health Services Res.

[41] Kuys SS, Burgess K, Fleming J, Varghese P, McPhail SM (2016). Evidence of Improved Efficiency in Functional Gains During Subacute Inpatient Rehabilitation. Am J Phys Med Rehabil.

[42] Drummond M, McGuire A. Economic evaluation in health care: merging theory with practice. Great Britain: Oxford University Press; 2004.

