# Supplementary figures and images for: Additional saturday occupational therapy for adults receiving inpatient physiotherapy rehabilitation: a prospective cohort study
Source: BMC Health Serv Res. 2022 May 9;22:617. doi: 10.1186/s12913-022-07727-7 (PMC9082956; doi:10.1186/s12913-022-07727-7)

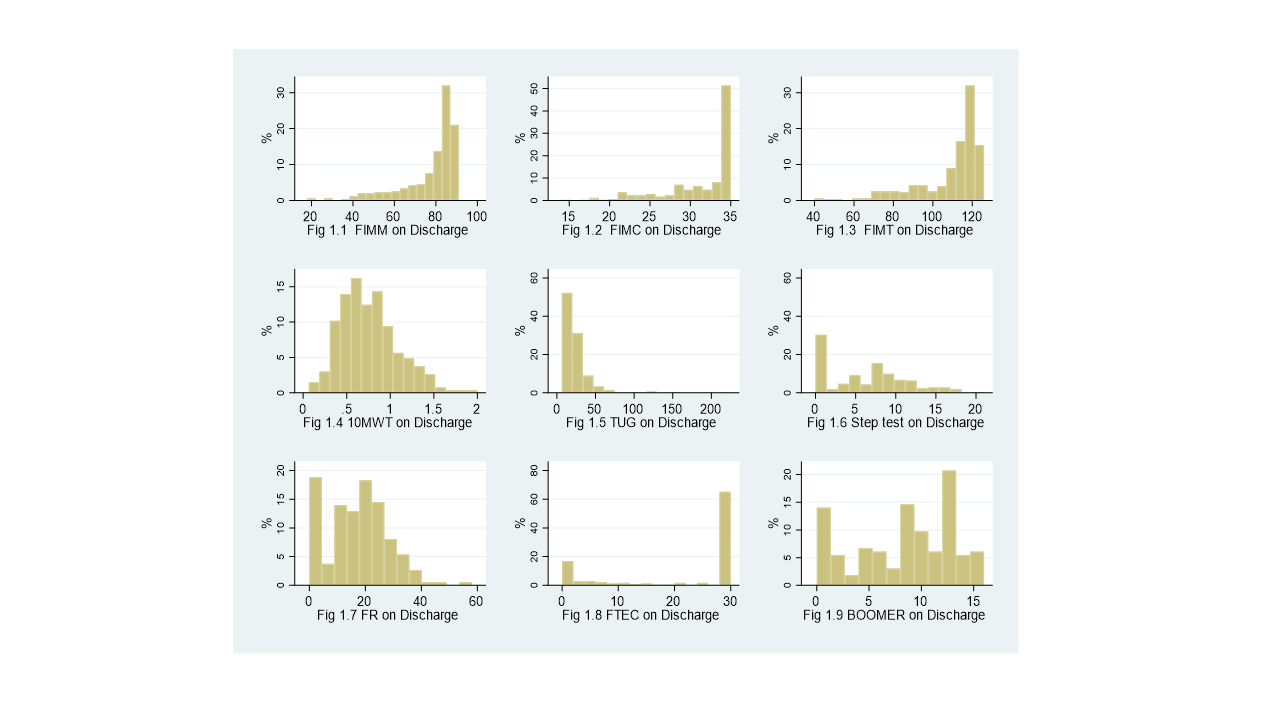

Supplement: Supplementary file 1 — Additional file 1: Figure A1.Distributions for nine measures of patient capability at discharge. [file 12913_2022_7727_MOESM1_ESM.png]
